# Are return to work beliefs, psychological well-being and perceived health related to return-to-work intentions among women on long-term sick leave for common mental disorders? A cross-sectional study based on the theory of planned behaviour

**DOI:** 10.1186/s12889-021-10562-w

**Published:** 2021-03-19

**Authors:** Åsa Hedlund, Marja-Leena Kristofferzon, Eva Boman, Annika Nilsson

**Affiliations:** 1 Department of Caring Sciences, University of Gävle, Kungsbäcksvägen 47, 801 76 Gävle, Sweden; 2 Department of Occupational Health and Psychology, University of Gävle, Gävle, Sweden

**Keywords:** Beliefs, Common mental disorders, Long-term sick leave, Return to work, Women

## Abstract

**Background:**

Long-term sick leave due to common mental disorders (CMDs) is a great burden in society today, especially among women. A strong intention to return to work (RTW) as well as symptom relief may facilitate RTW in this group. However, there is a lack of knowledge regarding what constitutes a strong intention. The Theory of Planned Behaviour is well-suited to identifying underlying beliefs about intentions to perform a behaviour. By including psychological well-being and perceived health, a more comprehensive picture of determinants of RTW intention might be achieved. Thus, the aim of the present study was to identify associations between RTW beliefs, psychological well-being, perceived health and RTW intentions among women on long-term sick leave due to CMDs, and to do so based on the Theory of Planned Behaviour.

**Methods:**

The study was cross-sectional. Between October 2019 and January 2020, 282 women on long-term sick leave (> 2 months) due to CMDs were included in the study. The questionnaires for data collection were: “RTW Beliefs Questionnaire”, the “General Health Questionnaire -12” and the “EuroQol Visual Analogue Scale”. Standard multiple regression analysis was performed both with and without adjustment for potential confounders.

**Results:**

The results showed that a more positive attitude towards RTW, stronger social pressure to RTW, higher perceived control over RTW and higher psychological well-being were associated with stronger RTW intention. The adjusted analysis eliminated the importance of psychological well-being for RTW intention, but showed that women who reported that their employer had taken actions to facilitate their RTW had stronger RTW intention.

**Conclusion:**

The RTW beliefs, derived from the Theory of Planned Behaviour, were all important for a strong RTW intention, while psychological well-being and perceived health showed weaker associations. Furthermore, having an employer that take actions for facilitating RTW was associated with stronger RTW intentions. Though some caution is warranted regarding the representativeness of the sample, the results do improve our understanding of some important determinants of RTW intention among women on long-term sick leave for CMDs.

**Supplementary Information:**

The online version contains supplementary material available at 10.1186/s12889-021-10562-w.

## Background

Common mental disorders (CMDs) is a leading cause of disability both internationally and in Sweden [1, 2]. Sick leaves due to CMDs are often longer than sick leaves in general and women are over-represented in this group [3, 4]. Long-term sick leave is associated with negative economic and health consequences [5]. It is, therefore, urgent to identify factors that facilitate return to work (RTW) in this group. Among individuals with CMDs, earlier RTW may be achieved by reducing the symptom burden from the CMD or increasing perceived health [6]. Support from the workplace is important as well [6]. However, to meet the growing challenges associated with increasing rates of long-term sick leave for CMDs, there is a need for investigation of RTW intention [7], i.e. its determinants as well as its importance for RTW. Subsequently, the present study is a first step in investigating RTW intention among women on long-term sick leave due to CMDs, by focusing on identification of determinants of RTW intention. The Theory of Planned Behaviour is well-suited to investigating intention and its determinants [8]. By investigating the relationship between RTW beliefs, psychological well-being, perceived health and RTW intentions, comprehensive knowledge about what constitutes RTW intention among women on long-term sick leave due to CMDs can be generated.

Long-term sick leave, i.e. sick leave > 2 months [9], due to CMDs has increased in Sweden during the past decade, primarily among women [3]. Long-term sick leave may entail negative consequences for health-related aspects of life such as sleep, leisure activities, lifestyle, habits (food, exercise, reading etc.) and psychological well-being [5]. Several diagnosis groups within the overarching category of CMDs (i.e. stress-related disorders, depression and anxiety disorders), result in sick leaves of longer duration than sick leaves in general. Exhaustion syndrome accounts for the longest sick leaves, approximately 6 months [2] or longer [10]. Women are especially vulnerable to stress-related disorders such as exhaustion syndrome [2]. One proposed explanation for women’s higher prevalence of CMDs is their role as head of household [11], especially after the first child is born [12]. Women have also been shown to more often experience a family-work conflict, i.e. difficulties balancing demands from home and demands from work [13]. There is also a suggestion that women are assessed differently by health care personnel and are therefore put on sick leave more easily [12]. All of these mentioned causes for CMDs may negatively affect women’s beliefs about RTW and research has shown that female gender is associated with delayed RTW among individuals with mental health disorders [14]. However, RTW is not only about work resumption, but also to stay at work [15]. Today, there is a lack of evidence concerning how RTW can be facilitated among these women. It is known that previous episodes of sick leave due to CMDs, co-morbidity, educational level, age, perceived health, hours of sleep per night and employers’ support affect RTW among individuals with CMDs [2, 6, 10]. Regarding current knowledge about RTW interventions, the most effective intervention among persons with CMDs is contact with the workplace during sick leave, preferably in combination with organizational changes, gradual RTW or psychological therapy [16]. This is part of what is called the Rehabilitation Chain in Sweden [17], where the employer is obligated to establish a RTW plan that is adjusted to the employee’s needs, if possible [18]. However, these interventions are not flexible enough to address needs related to all disorders among the CMDs [16], and long-term sick leave due to CMDs is still a growing problem in society. Therefore, new, preferably theory-driven [7, 19, 20] research in the area of CMDs and sick leave is called for. Regarding women, for whom evidence-based interventions are most urgent because they are the most vulnerable group, the lack of knowledge is even greater.

One overlooked aspect of RTW after long-term sick leave due to CMDs is the individual’s own intention to RTW [7], which has been shown to predict RTW among persons with musculoskeletal disorders [21]. The term “intention” is similar to, and even used synonymously with, the more common concept of “expectation” [21]. It is well known that RTW expectation does predict time to RTW among individuals on sick leave due to CMDs [7]. There has, however, been criticism of both the fact that RTW expectations are measured narrowly and differently in different studies, and of the fact that there is a knowledge gap regarding the basis for RTW expectations [7]. It is therefore suggested to move forward by investigating the broader concept of RTW intention in relation to RTW among individuals with CMDs, and to do so using a more consistent measurement based on theory [7]. An appropriate theory for doing this is the Theory of Planned Behaviour [8].

This theory is well-suited to investigating the intention to perform a behaviour and has been able to explain the importance of intention for various behaviours [22, 23], but rarely in relation to RTW. According to the Theory of Planned Behaviour [8], a strong intention to perform a behaviour leads to actual performance of the behaviour. Intention is defined as the degree to which a person is motivated or willing to perform a behaviour or his/hers readiness to execute the behaviour [8]. When measuring intention according to the Theory of Planned Behaviour, using a combination of items on intention, expectation and willingness to perform a behaviour is common [24].

Moreover, intention is based on underlying beliefs regarding the behaviour, which are essential to identify before predicting the actual behaviour [8]. These beliefs are three conceptually independent determinants: the individual’s own attitude towards, subjective norms (i.e., social pressure) and perceived behaviour control over a behaviour. Underlying these determinants are salient beliefs: behavioural beliefs (attitude), normative beliefs (subjective norm), and control beliefs (perceived behavioural control). Behavioural beliefs concern the advantages and disadvantages believed to be associated with the behaviour. Normative beliefs concern beliefs about important people in the individual’s life who will support or not support the performance of the behaviour. Control beliefs concern the believed barriers to and facilitators of performing the behaviour [24]. Dunstan et al. [21] found that behavioural beliefs, normative beliefs and control beliefs all showed a significant positive relationship with RTW intention among individuals with musculoskeletal disorders, which often co-occur with CMDs [25].

Regarding CMDs, they entail various physical and mental symptoms that decrease interest in things in life and the motivation to act [26]. A qualitative study, based on the Theory of Planned Behaviour and conducted on women on long-term sick leave due to CMDs, identified that symptoms from the CMD were perceived as barriers to RTW [27]. Based on this, it is reasonable to take psychological well-being and perceived health into account when identifying determinants of RTW intention among women on long-term sick leave due to CMDs. This is supported by the recommendation to include additional determinants that may increase the explanatory power of the intention, when using the Theory of Planned Behaviour [8].

Using the Theory of Planned Behaviour, including psychological well-being and perceived health, is a way of filling the gap in our knowledge about what underlies RTW intentions among women on long-term sick leave for CMDs. This provides a base for future prediction of actual RTW, as well as increasing our understanding of determinants of RTW intention in this group. Because women are most affected by long-term sick leave due to CMDs, there is value in studying them separately. This makes it easier to draw conclusions about the most vulnerable group, which is the most urgent to target. Thus, the present study aimed to identify associations between RTW beliefs, psychological well-being, perceived health and RTW intentions among women on long-term sick leave due to CMDs, and did so using the Theory of Planned Behaviour as its theoretical base.

## Methods

### Design

The study was theory driven, cross-sectional and correlational in design.

### Participants

In October 2019 and January 2020, a total of 1196 women from two regions in central Sweden were invited by the Social Insurance Agency (SIA) to participate in the study. To be included, the women had to meet the following criteria: a physician diagnosis within F32-F33, F35-F39 (mood disorders) and/or F40-F48 (neurotic, stress-related and somatoform syndromes), sick leave duration > 2 months and < 2 years (partial or full), age > 18 years and an understanding of written Swedish. If they were unemployed, they were not invited, as the target behaviour for the study was RTW. Of the 1196 women, 371 responded to the questionnaires, giving a response rate of 31%. However, 89 women were excluded because they were not on sick leave, were unemployed or returned an incomplete questionnaire. Hence, a total of 282 women were included in the study. A non-response analysis could not be performed because the researchers did not have access to SIA’s register of the women who declined to participate.

### Data collection

First, a physical meeting between the research group and the SIA took place to exchange information about the study and possible ways of identifying potential participants. Lists with the inclusion and exclusion criteria were prepared for the SIA to use when identifying potential participants. The lists, information letters and questionnaires were then handed over to the SIA. When the participants had answered the questionnaires, they send them back to the researchers in an enclosed and coded envelope. Two reminders were sent out, separated by a 2-week interval. Further description of the questionnaires is provided below.

### RTW beliefs questionnaire

The participants’ RTW beliefs were measured using the RTW Beliefs Questionnaire. The questionnaire was developed by the researchers for women on long-term sick-leave due to CMDs, was based on the Theory of Planned Behaviour and developed according to a manual written by Francis et al. [24]. The questionnaire consisted of 60 items divided into seven scales: four for general direct measures (intention, attitude, subjective norms, and perceived behavioural control) and three for behaviour-specific indirect measures: behavioural beliefs (indirect for attitude), normative beliefs (indirect subjective norm), and control beliefs (indirect perceived behavioural control). All items were formulated to suit women with CMDs who are on full-time or part-time sick leave, i.e. women who were on full-time sick leave focused on RTW and women that worked to some extent focused on staying at work when responding. Items for direct measures of RTW intentions were: “I expect to/want to/intend to …” ; for attitude: “RTW is for me: harmful-beneficial/good-bad/pleasant-unpleasant/worthless-useful”; for subjective norm: “It is expected of me that I should … /I feel under social pressure to … /People who are important to me want me to …” ; for perceived behavioural control: “I’m confident that I could … / for me RTW is (easy-difficult), The decision to RTW is beyond my control/Whether I RTW is entirely up to me”. However, the latter item from perceived behavioural control was removed due to low inter-item and item total correlation. All items for direct measures were rated on unipolar scales [1–7].

**Fig. 1:**
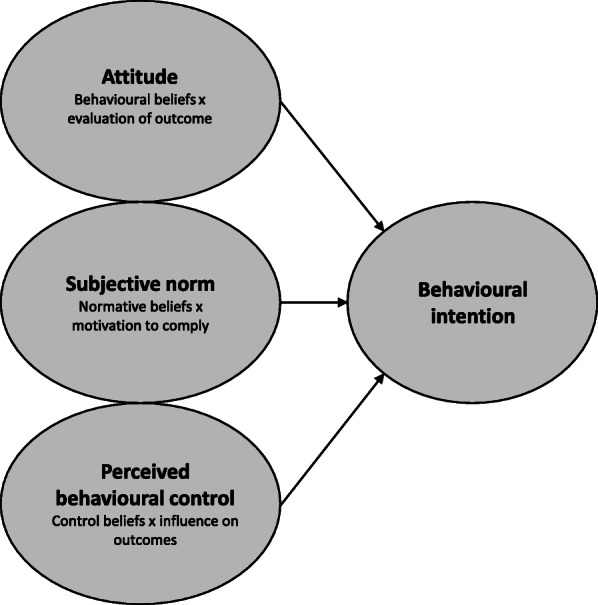
The Theory of Planned Behaviour with direct and indirect measures by Francis et al. [20]. The final step in the theory, behaviour, is not included in this figure

In an ‘elicitation study’, items for indirect measures were developed from interviews with 20 women with experience of long-term sick leave due to CMDs [24]. Based on the Theory of Planned Behaviour [24], behavioural beliefs are divided into the advantages and disadvantages of RTW, the subjective norm among supporters and nonsupporters of RTW and control beliefs in facilitators of and barriers to RTW. Inclusion of indirect measures is recommended because they provide the breadth of beliefs underlying RTW intention (and attitude, subjective norm and perceived behavioural control). This allows us to explain the variance in RTW intentions and its underlying determinants [28]. Half of the items for indirect measures were rated on unipolar scales [1–7], while the other half were rated on bipolar scales (− 3 to + 3). To prepare the data for analysis, each item for indirect measures was multiplied with an evaluation of outcome (behavioural beliefs), motivation to comply (normative beliefs), and influence on outcomes (control beliefs) to create item ranges between − 21 and + 21. Hence, indirect measures reveal the women’s believed importance of the advantages, disadvantages, supporters, nonsupporters, facilitators and barriers for RTW. Figure 1 shows the theory including direct and indirect scales. A total score for each of the seven scales was calculated. To confirm the validity of scales for the indirect measures [24], correlations (Spearman’s rank coefficient) between the indirect and direct scales were calculated and showed moderately positive correlations [29]: 0.55 (attitude), 0.39 (subjective norms) and 0.48 (perceived behavioural control). The internal consistency (Cronbach’s α) of the scales for the direct measures were: 0.92 (intention), 0.85 (attitude), 0.43 (subjective norms) and 0.60 (perceived behavioural control). A Cronbach’s alpha value of 0.60 is preferable according to the manual written by Francis et al. [24]. Questions about participant characteristics such as age, profession, number of children at home and sick leave history were included at the beginning of the questionnaire. Age, profession, number of children at home and hours of sleep were written on a blank line in the questionnaire. Co-morbidity, previous sick leave and the employer’s actions to facilitate the woman’s RTW had the response options yes – no. Level of physical exercise and education were measured on 4-point scales, where higher values meant more days of physical exercise and higher education. An English version of the RTW Questionnaire is provided as supplementary material.

### General health Questionnaire-12

To measure psychological well-being, the General Health Questionnaire-12 (GHQ-12) [30] was used; this is a commonly used instrument in research within occupational health [30]. It was developed in the 1970s to detect individuals with non-psychotic psychiatric disorders [31] and has since been reduced to create different versions. The GHQ-12 is one of the most popular versions due to its brevity; it consists of 12 items rated on a 4-point scale. This scale can be scored in a binary fashion (0–0–1-1) or scored as a Likert scale (0–1–2-3) [32]. In the present study, we used Likert scaling (0 = Never, 1 = Sometimes, 2 = Quite often, to 3 = Always), as we wanted a more sensitive measure. Items were for example “I feel I’m capable of making decisions” and “I can manage difficulties”. Before the analysis, the six items with a positive endpoint to the right were reversed so that the lower scores would indicate higher psychological well-being. The scores were then summed to a total score (range 0–36). There are various suggestions regarding where to put the cut-off point for this instrument [33]. However, a threshold of 12 is commonly recommended [34]. Hence, in the present study, a summed value > 12 is considered to indicate reduced psychological well-being. A systematic review has shown that the GHQ-12 has good internal consistency and structural validity, as well as excellent construct validity [35]. Internal consistency (Cronbach’s α) for the current sample was 0.89.

### EuroQol visual analogue scale

Perceived health was measured using the EuroQol Visual Analogue Scale (EQ-VAS) [36]. The scale ranges from 0 to 100, where 0 is the “worst imaginable health state” and 100 is the “best imaginable health state”. Participants were asked to mark on the scale where they perceived their current health to be. EQ-VAS is a widely used instrument and has shown excellent test-retest stability (reliability) as well as construct validity [37, 38]. Often, a cut-off value is not of interest when using this instrument, which is why it is difficult to find a cut-off value in the extant literature. However, a study on persons with heart problems used a cut-off point of 60, i.e. a score < 60 indicated poor health [39].

### Statistical analysis

All analyses were conducted in IBM SPSS statistics 24. Descriptive statistics were used to depict participant characteristics. Bivariate correlation analyses with Spearman’s rank coefficient were performed between items for indirect measures and total score for scales for direct measures. Two regression models were built, with RTW intention as the dependent variable. In the first model, attitude, subjective norm, perceived behavioural control, psychological well-being and perceived health were entered as independent variables. In the second model, potential confounders were added. The chosen potential confounders were: the employer’s actions to facilitate the woman’s RTW, age, previous episodes of sick leave due to CMDs, co-morbidity, educational level and hours of sleep per night. Previous research has indicated that these confounders may affect RTW [2, 6, 10]. The level of significance was set to *p* < 0.05. Correlations between the independent variables – calculated using the Pearson correlation coefficient for the continuous variables and the Phi coefficient for the binary variables – ranged from 0.00 (subjective norm and hours of sleep) to − 0.66 (psychological well-being and perceived health).

Assumptions for regression analysis, such as normality, multicollinearity, linearity and homoscedasticity [40], were largely met. No Variance Inflation Factor value exceeded 2.55, indicating no presence of multicollinearity [40]. However, most scales were slightly skewed, and the RTW intention scale showed a ceiling effect. Nonetheless, an examination of the residual plots in the regression analyses did not indicate any pronounced deviation from linearity, which is why this was not considered a major problem. The intention and subjective norm scales had a few minor outliers each. Removing those outliers did not yield substantially different results, and thus they were left in the analyses. Furthermore, the residual statistics in the regression analyses revealed no major problems with the outliers, as Cook’s Distance value was < 0.08, which is well under the critical limit of 1.0 [40]. Missing values within the scales did not exceed 2.5%, and overall, 1% of the values were missing. To deal with missing data, “exclude cases pairwise” was applied [41]. Because there is no existing evidence that intention predicts RTW among women on sick leave due to CMDs [7], regression analyses with only RTW expectation were conducted (see supplementary material, Table 1).

## Results

### Participant characteristics

The mean age was 45 (range 22–66 years). They reported sleeping 6.7 h a night (range 2–14) but needing 8.1 h (range 5.5–24). Furthermore, participant characteristics presented in Table 1 show that a majority of the women were born in Sweden and lived with a partner. Approximately half of the women had a university education. The single most common diagnosis was stress-related disorders and slightly less than half worked within the health/social care or school sector. Over a third perceived their economic situation to be unsatisfactory or very unsatisfactory, while approximately a fifth perceived it to be good or very good. The single most common frequency of physical activity was 1–3 days/week (> 30 min.). Over half of the women reported comorbidities, mostly musculoskeletal disorders. A majority were on part-time sick leave and reported that their employer had taken actions to facilitate their RTW process, and a majority had also previously been on sick leave due to CMDs.

**Table 1:** Self-reported characteristics of the participants (*N* = 282)

Variables	Frequency (%)
Country of birth	
Sweden	263 (93.3)
Other^a^	18 (6.4)
Not available	1 (0.3)
Have > 1 children living at home^b^	136 (48.2)
Have no children at home	142 (50.4)
Missing	4 (1.4)
Marital status	
Living with partner/parents^c^	207 (73.4)
Living alone with/without children	75 (26.6)
Education	
Elementary	27 (9.6)
Upper secondary	126 (44.7)
Post-upper secondary	4 (1.4)
University	125 (44.3)
Diagnosis	
Stress-related disorders	126 (44.6)
Depression	62 (22.0)
Anxiety	7 (2.5)
A combination of stress-related disorders and/or anxiety	
and/or depression	71 (25.2)
Other^d^	3 (1.1)
Missing	13 (4.6)
Profession	
Health care, schools and social service	140 (49.7)
Administrative work	40 (14.2)
Sales and service	33 (11.7)
Leading position or self-employed	27 (9.6)
Industry workers and engineers	11 (3.9)
Other^e^	31 (10.9)
Economy	
Very good/good	61 (21.6)
Acceptable	110 (39.0)
Unsatisfactory/very unsatisfactory	110 (39.0)
Missing	1 (0.4)
Days of physical exercise (> 30 min) per week	
0 days	46 (16.3)
1–3 days	155 (54.9)
4–5 days	38 (13.5)
6–7 days	40 (14.2)
Missing	3 (1.1)
Other health problems (comorbidity)	
Yes^f^	95 (33.7)
No other health problem except the CMD	185 (65.6)
Missing	2 (0.7)
Sick leave degree (%)	
100	106 (37.6)
25–75	176 (62.4)
Employer had taken actions to facilitate the woman’s RTW	
Yes^g^	174 (61.7)
No	103 (36.5)
Missing	5 (1.8)
Previous sick leave due to CMD	
Yes	166 (58.9)
No	116 (41.1)

^a^Half from Europe and a half from other parts of the world

^b^Almost half did not have any children

^c^Six lived apart with their partner. Two still lived with their parents

^d^Obsessive Compulsive Disorder

^e^E.g., craft or media jobs

^f^Most prevalent musculoskeletal disorders

^g^Mainly individual adaption of the working situation

### Descriptive statistics on the study variables

Table 2 shows that women reported low psychological well-being and perceived health. However, the women reported high RTW intentions, positive attitudes and strong social pressures towards RTW, while reporting lower levels of perceived behavioural control over RTW.

**Table 2:** Descriptive statistics on the dependent variable, independent variables and the underlying indirect measures (*N* = 282)

Variable	n	Scale range	Mean (SD)
Intention to RTW^a^	276	1–7^b^	5.5 (1.9)
Attitude	272	1–7^b^	4.7 (1.5)
*Behavioural beliefs x evaluation of outcome*	279	− 189 – 189^c^	104.6 (38.8)
Subjective norm	279	1–7^b^	4.8 (1.3)
*Normative beliefs x motivation to comply*	269	− 147- 147^c^	54.8 (35.7)
Perceived behavioural control	278	1–7^b^	3.9 (1.3)
*Control beliefs x influence on outcomes*	270	−147- 147^c^	- 1.6 (31.7)
Psychological well-being	268	0–36^d^	18.8 (6.8)
Perceived health	272	0–100^e^	46.4 (18.3)

^a^Return to work

^b^The higher the value, the more positive the attitude/stronger the social pressure/higher the perceived behavioural control

^c^Values > 0 indicate a positive attitude/strong social pressure/high perceived control to RTW

^d^The higher the value, the more reduced psychological well-being. Cut-off point = 12

^e^The higher the value, the better the perceived health. Cut-off point = 60

### Bivariate correlations between indirect measures and direct measures of RTW-beliefs

Bivariate correlations between items for indirect measures and total score for scales for direct measures are presented in Table 3. The items “improved health” and “meaningfulness” were strongest correlated with the direct scale for attitude. For the subjective norm, “family/relatives” showed the strongest correlation with its direct scale. Furthermore, “worsened health” and “well-adapted work tasks possible to perform at one’s own pace” were strongest correlated with the scale for direct measures of perceived behavioural control.

**Table 3:** Bivariate correlations^a^ between the indirect and direct measures (*n* = 271–278)

Items for indirect measures	Scales for direct measures^b^		
	Attitude	Subjective norm	Perceived behavioural control
*Behavioural beliefs*^*c*^			
*Advantages of RTW*			
Improved health	0.61**		
Meaningfulness	0.53**		
Better daily routines	0.47**		
Social context	0.46**		
Feeling of competence	0.45**		
Security	0.43**		
Improved personal economy	0.10		
*Disadvantages of RTW*			
Risk of increased symptoms	−0.41**		
Risk of performing worse than previously	0.14*		
*Normative beliefs*^*d*^			
*Supporters of RTW*			
Family/relatives		0.53**	
Friends		0.46**	
Social Insurance Agency		0.32**	
Employer		0.32**	
Co-workers		0.24**	
*Nonsupporters of RTW*			
Family/relatives		−0.12*	
Friends		−0.03	
*Control beliefs*^*e*^			
*Facilitators of RTW*			
Well-adapted work tasks I can perform at my own pace			0.35**
Support from friends, family and relatives			0.30**
Support from health care professionals			0.19**
*Barriers to RTW*			
Worsened health			0.48**
Employer, co-workers and/or authorities making unreasonable demands on my work performance			0.30**
Lack of support from those around me			0.19**
My own unreasonable demands on my work performance			0.17**

^a^Correlation with Spearman’s rank coefficient

^b^Higher values reflect more positive attitude to-, stronger social pressure to- and stronger perceived behavioural control of RTW

^c^Behavioural belief (e.g. If I RTW, my health will get better: strongly disagree (1)-strongly agree [7]) x evaluation of outcome (e.g. that my health gets better is for me: not important at all (−3) – very important (+ 3)). A higher value reflect a higher perceived importance of the advantages/disadvantages for RTW

^d^Normative beliefs (e.g. My friends want me to RTW: strongly disagree (− 3)-strongly agree [3]) x motivation to comply (e.g. That my friends want me to RTW is for me: not important at all (1)- very important [7]. A higher value reflect a higher perceived importance of the supporters/nonsupporters for RTW

^e^Control belief (e.g. That I get well adapted work tasks is: unlikely [1] – likely [7]) x influence on outcome (e.g. Well adapted work tasks makes it: more difficult (− 3) – easier (+ 3) to RTW). A higher value for facilitators reflect higher motivation to RTW based on the facilitator. Regarding barriers, a lower value reflects a stronger barrier, i.e. that it makes it more difficult to RTW according to the women

** Significant at the 0.01 level

* Significant at the 0.05 level

### Determinants of RTW intention

Table 4 show the results from the regression analyses, with RTW intention as the outcome variable. The results showed that women who had more positive attitudes towards RTW, perceived stronger social pressure to RTW, had stronger perceived behavioural control over RTW and higher psychological well-being, had stronger RTW intention. The model was significant (F (5,254) = 38.73, *p* < 0.001) and was able to explain 42% of the variance in RTW intention. When the analysis was adjusted for potential confounders, the results still showed that women who had a more positive attitude, felt stronger social pressure to RTW and had stronger perceived behavioural control to RTW had stronger RTW intention, but psychological well-being was no longer a significant variable. However, the adjusted model showed that women who reported that their employer had taken actions to facilitate their RTW had stronger RTW intentions. The model was significant (*F* = (11,248)19.79, *p* < 0.001) and explained 44% of the variance in RTW intention. In both the unadjusted and adjusted analysis, a positive attitude was by far the strongest determinant of RTW intention. The supplementary material (see Supplementary material: Table 1) shows the result of the regression analyses with RTW expectation as the dependent variable, which reveal the same pattern as the analyses with intention as the dependent variable.

**Table 4:** Multiple linear regression analysis with intention^a^ to RTW^b^ as the dependent variable (*n* = 259)

Variables	Unadjusted analysis		Adjusted analysis	
	β	*p-value*^*c*^	β	*p-value*^*c*^
Attitude^d^	**0.37**	**< 0.001**	**0.36**	**< 0.001**
Subjective norm^d^	**0.17**	**0.001**	**0.15**	**0.003**
Perceived behavioural control^d^	**0.19**	**0.002**	**0.15**	**0.016**
Psychological well-being^e^	**−0.14**	**0.046**	−0.08	0.289
Percieved health^e^	0.10	0.150	0.10	0.129
Employer actions^f^			**0.17**	**0.001**
Age			0.01	0.815
Previous sick leave^f^			−0.04	0.362
Comorbidity^f^			0.04	0.390
Education level^e^			0.06	0.225
Hours of sleep/night			0.03	0.527
Adjusted R^2^	**0.42**	**< 0.001**	**0.44**	**< 0.001**

^a^The higher the value, the stronger the intention

^b^Return to work

^c^Level of significance: *p* = < 0.05

^d^The higher the value, the stronger the intention/attitude/social pressure and perceived behavioural control

^e^The higher the value, the lower psychological well-being and higher perceived health and education level (continuous scales)

^f^Dichotomous variables where 0 = has not been on sick leave due to CMD before/ no comorbidity/employer has not taken any actions to facilitate the woman’s RTW. 1 = has been on sick leave due to CMD before/ presence of comorbidity/employer has taken actions to facilitate RTW

Note: Bold numbers represent significant values

## Discussion

The results showed that women had strong RTW intentions, but low psychological well-being and perceived health. They had positive attitudes towards and strong social pressure to RTW, but perceived relatively low control over it. Positive attitudes towards RTW, strong social pressure to RTW, high perceived behavioural control over RTW and high psychological well-being were associated with a stronger intention to RTW. However, when potential confounders were included in the analysis, psychological well-being was no longer significantly associated with RTW intention. Instead, women who reported that their employer had taken actions to facilitate their RTW had stronger RTW intention.

The result showed that a positive attitude towards RTW was the most important determinant of a strong RTW intention. This is in line with earlier research among individuals with musculoskeletal disorders [21]. In the present study, the correlations between indirect and direct measures (Table 3) showed that better health and meaningfulness were the strongest indirect measures within the attitude. This means that if the women believed that RTW would result in better health and a feeling of meaningfulness, and that this was important to them, they were more likely to have a positive attitude towards RTW. This emphasizes the importance of health-promoting workplaces, which is described by the World Health Organization as a priority setting for health promotion in the twenty-first century [42]. This involves, for example, meeting the needs of employees and developing the positive aspects of work as well as the strengths of the individual employees [43]. Based on this, it may be important to provide the women with work tasks that correspond not only to their current capacity, as described in the “Rehabilitation Chain” from the SIA [17], but also to their interests and ideas about what constitutes a meaningful job.

Furthermore, the results showed that women who perceived strong social pressure to RTW had higher RTW intentions. However, earlier research has shown that depression symptoms may increase when the individual is perceiving social pressure to feel better [44]. Table 3 shows that social pressure from family/relatives had the strongest correlation with the subjective norm, i.e., that pressure from the women’s family was most important to the feeling of social pressure to RTW. This is in line with findings showing that women more often experience a family-work conflict [13]. More research is needed to investigate the role of social pressure for RTW intention among women within different family constellations. For example, are there different determinants of RTW intention among single women than among married women with children?

Moreover, stronger perceived behavioural control over RTW was significantly associated with stronger RTW intention. Interestingly, the women in the present study had a job to return to (which might be seen as a part of *actual* control as described by Ajzen [8]), but according to the descriptive statistics in Table 2, they perceived relatively low control over RTW. This discrepancy may indicate that the women did not feel they had control over their CMD symptoms, which in turn affects the ability to RTW [6]. The indirect measures that were most strongly correlated with perceived behavioural control were the barrier “worsened health” upon RTW and the facilitator “having well-adapted working tasks that one can perform at one’s own pace”. One interpretation of this finding is that planning for and providing individualized work tasks are needed to reduce the barrier entailed in the belief that one’s health will worsen upon RTW.

Furthermore, women with high psychological well-being had stronger RTW intention. It is known that symptom burden is an important predictor of RTW among persons on sick leave due to CMDs, i.e. lower symptom severity is associated with earlier RTW [6]. However, when adding potential confounders to the analysis, psychological well-being was no longer significantly associated with RTW intention. Instead, the employer’s effort to facilitate the woman’s RTW emerged as a significant factor. In other words, women who reported that their employer had taken actions to facilitate RTW had stronger RTW intention. Previous research among persons with CMDs has shown similar results, indicating that employer support is an important factor for RTW [6]. Over a third of the women in the present study reported that their employer had not taken actions to facilitate their RTW. In Sweden, employers are obligated to plan RTW-facilitating adjustments for individuals on long-term sick leave (> 60 days) [18]. The plan is to be made together with the individual on sick leave. More research is needed to investigate why a large number of women reported a lack of facilitating actions on the part of their employer. Moreover, it is not clear whether it is the actions per se that are important, or whether it is the social support signified by such actions. Previous research has indicated that the social aspect of employer support is more important than characteristics of the work itself [45] as well as which aspects of employer actions matter for RTW intention.

Perceived health was not significantly associated with RTW intention in the present study. One explanation may be the high correlation between psychological well-being and perceived health in the analysis. It is important to keep in mind that, in the present study, the most crucial aspect of having a positive attitude towards RTW among the women was the belief that their health would improve upon RTW. Hence, current perceived health seems to be less important to RTW intention than is the belief that one’s health will improve in the future.

In summary, using the Theory of Planned Behaviour, a range of determinants of RTW intentions have been identified. This is relevant to health care professionals, employers and other stakeholders involved in the RTW process. However, the relationship between RTW intention and actual RTW among individuals on long-term sick leave due to CMDs is not known, but it is well known that RTW expectation does predict actual RTW in this group [7]. Supplementary material in the current study reveals that RTW expectation shows the same pattern as RTW intention, which makes it reasonable to assume that RTW intention is relevant for actual RTW as well. Moreover, because this is a cross-sectional study, no cause-and-effect conclusions can be drawn. Longitudinal research is the next natural step in exploring the importance of RTW beliefs, psychological well-being and perceived health for actual RTW. Furthermore, additional psychometric properties of the RTW Beliefs Questionnaire must be tested to evaluate the instrument’s validity and reliability in more detail.

### Methodological considerations

The present study has some limitations. First, it had a relatively small sample size and a low response rate. This may be due to symptoms related to CMDs; i.e., concentration difficulties or feelings of exhaustion may make completing comprehensive questionnaires challenging. Because of this, we may have missed the women most affected by CMD symptoms. One strength of the selection process was that participants were selected based on a diagnosis issued by a physician and not on the women’s own opinions about why they were on sick leave. However, the sample may be considered heterogeneous due to the varying degree of sick leave (25–100%). It is reasonable to assume that there are differences in RTW beliefs between women who are on full-time sick leave and those who work to some extent. However, because the target behaviour of the present study was RTW, we argue that it was appropriate to include all of them. The RTW Beliefs Questionnaire is a new, theory-based instrument, developed especially for women who are on sick leave for CMDs. To our knowledge, no similar instrument exists, and thus it is considered to make a valuable contribution to the field. However, it needs to be tested further regarding its psychometric properties. What we know thus far is that one of the sub-scales, subjective norm, showed poor internal consistency. If this had been a more stable construct, the results might have given a more accurate picture of its association with RTW intention. However, the results were in line with previous research [21], showing that subjective norm was positively associated with RTW intention, which is why we believe the overall results are valid. Further research is needed to evaluate the problem with the scale of a subjective norm among women on long-term sick leave due to CMDs, i.e., why it is heterogenous and how the scale should be used most accurately. For example, social pressure from “important people” may be more crucial to RTW intention than is social pressure from undefined “others”. Furthermore, the representativeness of the sample may be questioned. Unfortunately, a non-response analysis was not possible to perform, but nationally, sick leave > 14 days due to mental disorders among women is most common in the age group 35–44 years [2]. The mean age in the present study was somewhat higher, which calls into question the generalizability of the results. Moreover, the sampling was done in a rural area in Sweden with no big cities, which may negatively affect generalizability, as there are different kinds of social support and surroundings in big cities that may influence the experience of being on sick-leave due to CMDs [1]. However, a large proportion of the included women worked in the health or social care sector, which is in line with the occupations of women on sick leave for CMDs nationally [2]. Caution is in order when generalizing to persons not born in Sweden, however, as only 7% of the women in the present study were born abroad.

## Conclusion

The RTW beliefs derived from the Theory of Planned Behaviour were all important to having a strong RTW intention, while psychological well-being and perceived health showed weaker associations. This supports the notion that the Theory of Planned Behaviour is a useful theoretical framework for use in RTW research. Overall, having a positive attitude towards RTW and an employer that takes actions to facilitate RTW were associated with stronger RTW intentions. This is valuable knowledge for health care professionals, employers and other stakeholders involved in women’s RTW process. Some caution is warranted, however, regarding the representativeness of the sample and interpretation of the results on the subjective norm. Despite some weaknesses in the new instrument measuring women’s beliefs about RTW, the study provided important indicators of which aspects may matter for RTW intentions among women on long-term sick leave due to CMDs. Naturally, as the present study is only a first step, more research is needed.

## Supplementary Information

**Additional file 1: Supplementary material, Table 1.**

**Additional file 2: Supplementary material**, RTW Beliefs Questionnaire

## Data Availability

The datasets used and/or analysed during the current study are available from the corresponding author on reasonable request.

## Abbreviations

CMDs: Common mental disorders
RTW: Return to work
SIA: Social insurance agency

## References

[1] World Health Organization. Depression and other Common Mental Disorders: Global Health Estimates. Available from: https://apps.who.int/iris/bitstream/handle/10665/254610/WHO-MSD-MER-2017.2-eng.pdf.

[2] Försäkringskassan (Social Insurance Agency). Socialförsäkringsrapport. Sjukfrånvaro i psykiatriska diagnoser. En registerstudie av Sveriges arbetande befolkning i åldern 20–69 år/Social Insurance Report. Sickness absence for psychiatric disorders. A register study of Sweden’s working population aged [Internet]. 2020 [cited 2020 Dec 16]. Available from: https://www.forsakringskassan.se/wps/wcm/connect/e12b777c-e98a-488d-998f-501e621f4714/socialforsakringsrapport-2020-8.pdf? MOD=AJPERES&CVID.

[3] Vingård E. Psykisk ohälsa, arbetsliv och sjukfrånvaro. En kunskapsöversikt/Mental illness, working life and sickness absence. An overview of current knowledge. A report from FORTE (Swedish Research Council for Health, Working life and Welfare) [Internet]. 2020 [cited 2020 Dec 17]. Available from: https://forte.se/publikation/psykisk-ohalsa-arbetsliv-och-sjukfranvaro/

[4] EU. Sick pay and sickness benefit schemes in the European UnionBackground reportfor the Social Protection Committee’sIn-Depth Review on sickness benefits Brussels, 17 October 2016 [Internet]. 2016. Available from: https://op.europa.eu/en/publication-detail/-/publication/fc7a58b4-2599-11e7-ab65-01aa75ed71a1/language-en.

[5] Floderus B, Göransson S, Alexanderson K, Aronsson G (2005). Self-estimated life situation in patients on long-term sick leave. J Rehabil Med.

[6] de Vries H, Fishta A, Weikert B, Rodriguez Sanchez A, Wegewitz U (2018). Determinants of sickness absence and return to work among employees with common mental disorders: a scoping review. J Occup Rehabil.

[7] Hedlund Å. An overlooked concept? Intention to return to work among individuals on sick leave due to common mental disorders: A scoping review. Health Soc Care Community. 2021;hsc.13293. [cited 2021 Jan 31] Available from: https://onlinelibrary.wiley.com/doi/10.1111/hsc.13293 10.1111/hsc.13293 33506561

[8] Ajzen I (1991). The theory of planned behavior. Organ Behav Hum Decis Process.

[9] Lidwall U. Långtidssjukskrivna – demografi, arbete, yrke, diagnos, sjukpenningrätt och återgång i arbete 2003, 2005 och 2006/Persons on long-term sick leave - demography, work, profession and return to work 2003, 2005 and 2006 [Internet]. Stockholm; 2007. Available from: https://www.forsakringskassan.se/wps/wcm/connect/9e713747-85a1-4574-a4d3-6e6e422ef729/redovisar_2007_06.pdf? MOD=AJPERES

[10] Ulrik Lidwall & Christina Olsson-Bohlin. Lång väg tillbaka till arbete vid sjukskrivning. Korta analyser 2017:1/Long way back to work after sick leave. Short analyzes 2017:1 (Report from the Social Insurance Agency in Sweden) [Internet]. 2017. Available from: https://www.forsakringskassan.se/wps/wcm/connect/d57be02c-46dc-4079-b68d-760739441f11/korta-analyser-2017-1.pdf? MOD=AJPERES&CVID=.

[11] Chandra PS, Satyanarayana VA (2010). Gender disadvantage and common mental disorders in women. Int Rev Psychiatry.

[12] Hägglund SL& P. Jämställd sjukfrånvaro – Bedöms män och kvinnor likvärdigt/Equal sickness absence - are men and women assessed equally? (RiR 2019:19) (Report from Riksrevisionen/the Swedish NAO) [Internet]. Stockholm; 2019. Available from: https://www.riksrevisionen.se/rapporter/granskningsrapporter/2019/jamstalld-sjukfranvaro%2D%2D-bedoms-man-och-kvinnor-likvardigt-i-sjukskrivningsprocessen.html

[13] Stressforskningsinstitutet/Stress Research Institute. Arbete och självrapporterad hälsa bland svenska kvinnor och män/Work and self-reported health among Swedish women and men [Internet]. Stockholm; 2015. Available from: https://www.stressforskning.su.se/polopoly_fs/1.230061.1427290025!/menu/standard/file/Kvinnorochmänshälsawebb.pdf

[14] Prang KH, Bohensky M, Smith P, Collie A. Return to work outcomes for workers with mental health conditions: A retrospective cohort study. Injury. 2016;47(1):257–265. [cited 2020 Dec 17] Available from: http://dx.doi.org/10.1016/j.injury.2015.09.011 10.1016/j.injury.2015.09.011 26489393

[15] Steenstra IA, Lee H, De Vroome EMM, Busse JW, Hogg-Johnson SJ (2012). Comparing current definitions of return to work: A measurement approach. J Occup Rehabil.

[16] Mikkelsen MB, Rosholm M (2018). Systematic review and meta-analysis of interventions aimed at enhancing return to work for sick-listed workers with common mental disorders, stress-related disorders, somatoform disorders and personality disorders. Occup Environ Med.

[17] Försäkringskassan (Social Insurance Agency). Rehabiliteringskedjan/The rehabilitation chain [Internet]. 2020. Available from: https://www.forsakringskassan.se/omfk/vart_uppdrag/om_socialforsakringen/rehabiliteringskedjan

[18] Försäkringskassan (Social Insurance Agency). Plan för återgång i arbete/Plan for return to work [Internet]. 2020 [cited 2020 May 7]. Available from: https://www.forsakringskassan.se/!ut/p/z0/VcwxDsIwDAXQszBkDAliY6sQF4Cl6lI5NA0mrRM5bhG3J6q6sPl_vW_TmdZ0BCsGEEwEU83t5zlfMshLnc6NstdE4kket_7uS05UcPXKAjsvJeAKXNOY2LtvCLq8lzgyUK1TrRlLrEDPftgG-60XSRFEOxwYgrJ5AtL1hwbxHICCRr15L8oWHIBKz4tjjMd_ipXu0OTYHH6mNRjz/

[19] Nigatu YT, Liu Y, Uppal M, McKinney S, Gillis K, Rao S, Wang JL (2017). Prognostic factors for return to work of employees with common mental disorders: a meta-analysis of cohort studies. Soc Psychiatry Psychiatr Epidemiol.

[20] Andersen MF, Nielsen KM, Brinkmann S (2012). Meta-synthesis of qualitative research on return to work among employees with common mental disorders. Scand J Work Env Heal.

[21] Dunstan DA, Covic T, Tyson GA (2013). What leads to the expectation to return to work? Insights from a theory of planned behavior (TPB) model of future work outcomes. Work.

[22] Cooke R, Dahdah M, Norman P, French DP (2016). How well does the theory of planned behaviour predict alcohol consumption? A systematic review and meta-analysis. Health Psychol Rev.

[23] McDermott MS, Oliver M, Simnadis T, Beck EJ, Coltman T, Iverson D, et al. The theory of planned behaviour and dietary patterns: a systematic review and meta-analysis [Internet]. Prev Med. 2015;81:150–6 [cited 2020 Sep 6] Available from: https://pubmed.ncbi.nlm.nih.gov/26348455/ Academic Press Inc. 10.1016/j.ypmed.2015.08.020 26348455

[24] Francis, J., Eccles, M. P., Johnston, M., Walker, A. E., Grimshaw, J. M., Foy, R., Kaner, E. F. S., Smith, L. and Bonetti D. Constructing questionnaires based on the theory of planned behaviour: A manual for health services researchers. [Internet]. City Research Online. 2004. Available from: https://openaccess.city.ac.uk/id/eprint/1735

[25] Goesling J, Lin LA CD. Psychiatry and pain management: at the intersection of chronic pain and mental health. Curr Psychiatry Rep 2018;20(2):12, DOI: 10.1007/s11920-018-0872-4. 10.1007/s11920-018-0872-4 29504088

[26] Läkemedelsverket/Swedish Medical Products. Läkemedelsboken - Förstämningssyndrom, ångest och oro/Book of medical products - Mood disorders, anxiety and worry. [Internet]. 2017. Available from: https://lakemedelsboken.se/

[27] Hedlund Å, Boman E, Kristofferzon ML, Nilsson A. Beliefs about return to work among women during/after long-term sick leave for common mental disorders: a qualitative study based on the theory of planned behaviour. J Occup Rehabil. 2021. 10.1007/s10926-020-09946-3. Epub ahead of print. 10.1007/s10926-020-09946-3 PMC8298344 33492634

[28] Francis JJ, Johnston M, Eccles MP, Grimshaw J, Kaner EFS. Appendix C: Discussion paper. Measurement issues in the Theory of Planned Behaviour. A supplement to the Manual for constructing questionnaires based on the Theory of Planned Behaviour. 2004.

[29] Akoglu H (2018). User’s guide to correlation coefficients. Turkish J Emerg Med.

[30] Sconfienza C. Mätning av psykiskt välbefinnande bland ungdomar i Sverige. Användning av GHQ-12/Measurement of psychological well-being among adolescents in Sweden. Use of GHQ-12. [Internet]. Arbetslivsinstitutet/National Institute of Working Life. Stockholm; 1998. Available from: https://gupea.ub.gu.se/bitstream/2077/4181/1/ah1998_22.pdf

[31] Goldberg DP (1972). The detection of psychiatric illness by questionnaire. A technique for the identification and assessment of non-psychotic psychiatric illness.

[32] Holi MM, Marttunen M, Aalberg V (2003). Comparison of the GHQ-36, the GHQ-12 and the SCL-90 as psychiatric screening instruments in the Finnish population. Nord J Psychiatry.

[33] Goldberg DP, Oldehinkel T, Ormel J (1998). Why GHQ threshold varies from one place to another. Psychol Med.

[34] Child, Health and Parenting (CHAP) and Uppdrag psykisk ohälsa/Mission Mental Illness. GHQ-12 General Health Questionnaire-12 [Internet]. Available from: https://docplayer.se/110078292-Ghq-12-general-health-questionnaire-12.html.

[35] Bentley N, Hartley S, Bucci S (2019). Systematic review of self-report measures of general mental health and wellbeing in adolescent mental health. Clin Child Fam Psychol Rev.

[36] EuroQol office. EQ-5D [Internet]. 2020. Available from: https://euroqol.org/

[37] Wang HM, Patrick DL, Edwards TC, Skalicky AM, Zeng HY, Gu WW (2012). Validation of the EQ-5D in a general population sample in urban China. Qual Life Res.

[38] Khoudri I, Belayachi J, Dendane T, Abidi K, Madani N, Zekraoui A, Zeggwagh AA, Abouqal R (2012). Measuring quality of life after intensive care using the Arabic version for Morocco of the EuroQol 5 dimensions. BMC Res Notes.

[39] Lenzen MJ, WJM SOR, Pedersen SS, Boersma E, Maier W, Widimsky P (2007). The additional value of patient-reported health status in predicting 1-year mortality after invasive coronary procedures: a report from the euro heart survey on coronary revascularisation. Heart.

[40] Pallant J (2016). SPSS Survival Manual.

[41] Newman DA. Missing data: five practical guidelines. Organ Res Methods. 2014;17(4):372–411. Available from. 2014: https://doi.org/10.1177/1094428114548590

[42] World Health Organization. WHO | Workplace health promotion [Internet]. 2020 [cited 2020 Dec 11]. Available from: https://www.who.int/occupational_health/topics/workplace/en/

[43] World Health Organization. WHO | Mental health in the workplace. WHO [Internet]. 2019 [cited 2020 May 27]; Available from: https://www.who.int/teams/mental-health-and-substance-use/mental-health-in-the-workplace

[44] Dejonckheere E, Bastian B, Fried EI, Murphy SC, Kuppens P (2017). Perceiving social pressure not to feel negative predicts depressive symptoms in daily life. Depress Anxiety.

[45] Vemer P, Bouwmans C, Zijlstra-Vlasveld M. Let’s get back to work: survival analysis on the return-to-work after depression. 2013; Available from: http://dvpr.es/19fSI1U 10.2147/NDT.S49883 PMC3810438 24187499

[46] World Medical Association. WMA declaration of Helsinki – ethical principles for scientific requirements and research protocols. Fortaleza: 64th WMA General Assembly; 2013. Available from: https://www.wma.net/policies- post/wma-declaration-of-helsinki-ethical-principles-for-medicalresearch-involving-human-subjects/.

