# Supplementary material for: Are return to work beliefs, psychological well-being and perceived health related to return-to-work intentions among women on long-term sick leave for common mental disorders? A cross-sectional study based on the theory of planned behaviour
Source: BMC Public Health. 2021 Mar 19;21:535. doi: 10.1186/s12889-021-10562-w (PMC7977300; doi:10.1186/s12889-021-10562-w)
Supplement: Supplementary file 1 — Additional file 1: Supplementary material, Table 1. [file 12889_2021_10562_MOESM1_ESM.docx]

**Supplementary material**

**Table 1.** Multiple linear regression analysis with expectation^a^ of RTW^b^ as the dependent variable (n = 259)

| **Variables** | **Unadjusted analysis** | | **Adjusted analysis** | |
| --- | --- | --- | --- | --- |
|  | β | *p-value^c^* | β | *p-value^c^* |
| Attitude^d^ | **0.16** | **0.016** | **0.17** | **0.013** |
| Subjective norm^d^ | **0.20** | **0.001** | **0.17** | **0.006** |
| Perceived behavioural control^d^ | **0.24** | **0.001** | **0.21** | **0.004** |
| Psychological well-being^e^ | -0.12 | 0.139 | -0.05 | 0.536 |
| Percieved health^e^ | -0.03 | 0.661 | -0.03 | 0.709 |
| Employer actions^f^ |  |  | **0.13** | **0.038** |
| Age |  |  | -0.05 | 0.441 |
| Previous sick leave^f^ |  |  | -0.05 | 0.358 |
| Comorbidity^f^ |  |  | 0.02 | 0.682 |
| Education level^e^ |  |  | 0.08 | 0.175 |
| Hours of sleep/night |  |  | 0.06 | 0.359 |
| Adjusted R^2^ | **0.20**^g^ | **<0.001** | **0.21**^h^ | **<0.001** |

^a. The higher the value, the stronger the expectation (measured with a single item)^

^b. Return to work.^

^c. Level of significance:^ *^p^*^=<0.05^

^d. The higher the value, the stronger the intention/attitude/social pressure and perceived behavioural control.^

^e. The higher the value, the higher the psychological well-being, perceived health and education level (continuous scales)^

^f. Dichotomous variables where 0 = has not been on sick leave due to CMD before/ no comorbidity/employer has not taken any actions to facilitate the woman’s RTW. 1 = has been on sick leave due to CMD before/ presence of comorbidity/employer has taken actions to facilitate RTW.^

^Note: Bold numbers represent significant values^

^g. (F(5,254)=13.75,^ *^p^*^<0.001)^

^h. (F(11,248)=7.34,^ *^p^*^<0.001)^
