# Supplementary material for: Are return to work beliefs, psychological well-being and perceived health related to return-to-work intentions among women on long-term sick leave for common mental disorders? A cross-sectional study based on the theory of planned behaviour
Source: BMC Public Health. 2021 Mar 19;21:535. doi: 10.1186/s12889-021-10562-w (PMC7977300; doi:10.1186/s12889-021-10562-w)
Supplement: Supplementary file 2 — Additional file 2: Supplementary material, RTW Beliefs Questionnaire [file 12889_2021_10562_MOESM2_ESM.docx]

**RTW Beliefs Questionnaire**

**For those who are on full-time sick leave**

1. I expect to return to work within 3 months

Strongly disagree 1 2 3 4 5 6 7 Strongly agree

1. I want to return to work within 3 months

Strongly disagree 1 2 3 4 5 6 7 Strongly agree

1. I intend to return to work within 3 months

Strongly disagree 1 2 3 4 5 6 7 Strongly agree

**For those in work**

1. I expect to stay at work 3 months from now

Strongly disagree 1 2 3 4 5 6 7 Strongly agree

1. I want to stay at work 3 months from now

Strongly disagree 1 2 3 4 5 6 7 Strongly agree

1. I intend to stay at work 3 months from now

Strongly disagree 1 2 3 4 5 6 7 Strongly agree

**The statements below are aimed both at those on full-time sick leave and those currently in work. If you are on full-time sick leave, please focus on the phrase ”return to work”. If you are in work, please focus on the phrase ”stay at work”.**

1. If I return to work/stay at work, I will feel more like I am part of a social context.

Strongly disagree 1 2 3 4 5 6 7 Strongly agree

**8.** If I return to work/stay at work, I will have improved daily routines.

Strongly disagree 1 2 3 4 5 6 7 Strongly agree

**9.** If I return to work/stay at work, life will feel more meaningful.

Strongly disagree 1 2 3 4 5 6 7 Strongly agree

**10.** That my family/relatives want me to return to work/stay at work is for me:

Not important at all 1 2 3 4 5 6 7 Very important

**11.** That my friends want me to return to work/stay at work is for me:

Not important at all 1 2 3 4 5 6 7 Very important

**12.** That the Social Insurance Agency wants me to return to work/stay at work is for me:

Not important at all 1 2 3 4 5 6 7 Very important

**13.** If I return to work/stay at work, I feel that my health will improve.

Strongly disagree 1 2 3 4 5 6 7 Strongly agree

**14.** If I return to work/stay at work, there is a risk that my symptoms will increase.

Strongly disagree 1 2 3 4 5 6 7 Strongly agree

**15.** If I return to work/stay at work, there is a risk that I won’t manage the same achievements as before.

Strongly disagree 1 2 3 4 5 6 7 Strongly agree

**16.** That my colleagues want me to return to work/stay at work is for me:

Not important at all 1 2 3 4 5 6 7 Very important

**17.** That my employer wants me to return to work/stay at work is for me:

Not important at all 1 2 3 4 5 6 7 Very important

**18.**  That my family/relatives don’t want me to return to work/stay at work if there is a risk that my health will deteriorate is for me:

Not important at all 1 2 3 4 5 6 7 Very important

**19.** That I will receive support from my surroundings (friends, family and relatives) to return to work/stay at work is:

Unlikely 1 2 3 4 5 6 7 Likely

**20.** That I will receive support from healthcare staff (such as physician, counsellor, nurse) to return to work/stay at work is:

Unlikely 1 2 3 4 5 6 7 Likely

**21.** That I will be given well-adapted work tasks which I can perform at my own pace is:

Unlikely 1 2 3 4 5 6 7 Likely

**22.** Return to work/stay at work is for me:

Harmful 1 2 3 4 5 6 7 Beneficial

Worthless 1 2 3 4 5 6 7 Useful

Good 1 2 3 4 5 6 7 Bad

Pleasant 1 2 3 4 5 6 7 Unpleasant

**23.** Return to work/stay at work is for me:

Easy 1 2 3 4 5 6 7 Difficult

**24.** Increased participation in a social context is for me:

Not important at all -3 -2 -1 0 +1 +2 +3 Very important

**25.** Having better daily routines is for me:

Not important at all -3 -2 -1 0 +1 +2 +3 Very important

**26.** Perceiving meaningfulness in life is for me:

Not important at all -3 -2 -1 0 +1 +2 +3 Very important

**27.** That my health improves is for me:

Not important at all -3 -2 -1 0 +1 +2 +3 Very important

**28.** Being able to reach the same achievements as before at work is for me:

Not important at all -3 -2 -1 0 +1 +2 +3 Very important

**29.** Improving my personal economy is for me:

Not important at all -3 -2 -1 0 +1 +2 +3 Very important

**30.** Feeling secure in my place of work is for me:

Not important at all -3 -2 -1 0 +1 +2 +3 Very important

**31.** Feeling competent in my work is:

Not important at all -3 -2 -1 0 +1 +2 +3 Very important

**32.** Not experiencing an increase in my symptoms is for me:

Not important at all -3 -2 -1 0 +1 +2 +3 Very important

**33.** It is expected of me to return to work/stay at work.

Strongly disagree 1 2 3 4 5 6 7 Strongly agree

**34.** That my friends don’t want me to return to work/stay at work if there is a risk that my health will deteriorate is for me:

Not important at all 1 2 3 4 5 6 7 Very important

**35.** I am confident that I can return to work/stay at work if I want to.

Strongly disagree 1 2 3 4 5 6 7 Strongly agree

**36.** The decision to return to work/stay at work is beyond my control.

Strongly disagree 1 2 3 4 5 6 7 Strongly agree

**37.** Whether I return to work/stay at work is entirely up to me.

Strongly disagree 1 2 3 4 5 6 7 Strongly agree

**38.** If I return to work/stay at work, my economy will improve.

Strongly disagree 1 2 3 4 5 6 7 Strongly agree

**39.** If I return to/stay at the same workplace, I will feel secure.

Strongly disagree 1 2 3 4 5 6 7 Strongly agree

**40.** If I return to work/ stay at work I will keep/increase my sense of competence.

Strongly disagree 1 2 3 4 5 6 7 Strongly agree

**41.** That the employer, colleagues and/or authorities put unreasonable demands on my work performance is:

Unlikely 1 2 3 4 5 6 7 Likely

**42.** That I myself will put unreasonable demands on my work performance is:

Unlikely 1 2 3 4 5 6 7 Likely

**43.** That I will experience lack of support from my surroundings regarding returning to work/stay at work is:

Unlikely 1 2 3 4 5 6 7 Likely

**44.** That my health will deteriorate if I return to work/stay at work is:

Unlikely 1 2 3 4 5 6 7 Likely

**45.** I feel social pressure from my surroundings to return to work/stay at work.

Strongly disagree 1 2 3 4 5 6 7 Strongly agree

**46.** People who are important to me want me to return to work/stay at work.

Strongly disagree 1 2 3 4 5 6 7 Strongly agree

**47.** My family/relatives want me to return to work/stay at work.

Strongly disagree -3 -2 -1 0 +1 +2 +3 Strongly agree

**48.** My friends want me to return to work/stay at work.

Strongly disagree -3 -2 -1 0 +1 +2 +3 Strongly agree

**49.** My family/relatives do not want me to return to work/stay at work if there is a risk that my health will deteriorate.

Strongly disagree -3 -2 -1 0 +1 +2 +3 Strongly agree

**50.** My colleagues want me to return to work/stay at work.

Strongly disagree -3 -2 -1 0 +1 +2 +3 Strongly agree

**51.** The Social Insurance Agency wants me to return to work/stay at work.

Strongly disagree -3 -2 -1 0 +1 +2 +3 Strongly agree

**52.** My employer wants me to return to work/stay at work.

Strongly disagree -3 -2 -1 0 +1 +2 +3 Strongly agree

**53.** My friends do not want me to return to work/stay at work if there is a risk that my health will deteriorate.

Strongly disagree -3 -2 -1 0 +1 +2 +3 Strongly agree

**54.** Support from healthcare staff (such as physician, counsellor, nurse) makes me:
 Less motivated -3 -2 -1 0 +1 +2 +3 More motivated

to return to work/stay at work.

**55.** Well-adapted work tasks I can perform at my own pace make me:

Less motivated -3 -2 -1 0 +1 +2 +3 More motivated

to return to work/stay at work.

**56.** The employer’s, colleagues’ and/or authorities’ unreasonable demands on my work performance make it:

More difficult -3 -2 -1 0 +1 +2 +3 Easier

to return to work/stay at work.

**57.** My own unreasonable demands on my work performance make it:

More difficult -3 -2 -1 0 +1 +2 +3 Easier

for me to return to work/stay at work.

**58.** Lack of support from my surroundings makes it:

More difficult -3 -2 -1 0 +1 +2 +3 Easier

for me to return to work/stay at work.

**59.** Deterioration in my health makes it:

More difficult -3 -2 -1 0 +1 +2 +3 Easier

for me to return to work/stay at work.

**60.** Support from my surroundings (friends, family and relatives) makes me:

Less motivated -3 -2 -1 0 +1 +2 +3 More motivated

to return to work/stay at work.
